# The Social Malady

**Published:** 1913-12

**Authors:** Theo. Y. Hull

**Affiliations:** Secretary State Society of Social Hygiene, San Antonio, Texas


### Department of Eugenics.

Conducted by Dr. Malone Duggan and Dr. Theo. Y. Hull.

### THE TEXAS STATE SOCIETY OF SOCIAL HYGIENE.

Headquarters: San Antonio, Texas.

Dr. Malone Duggan, President, San Antonio.
Dr. F. E. Daniel, 1st Vice President, Austin.
Prof. A. Caswell Ellis, 2d Vice President, Austin.
Mrs. Eli Hertzberg, 3d Vice President, San Antonio.
Dr. Theo. Y. Hull, Secretary, San Antonio.
Mr. W. L. Evans, Treasurer, San Antonio.

EXECUTIVE COMMITTEE.

Dr. Malone Duggan, Chm.
Dr. Theo. Y. Hull, Sec'y.
Dr. W. F. McCaleb.
Hon. C. H. Jenkins.
Rev. A. W. S. Garden.
Hon. J. C. Willacy.
Mr. W. L. Evans.
Dr. Frank D. Boyd.
Dr. J. R. Nichols.
Dr. J. M. McCutchan.
Dr. W. A. King.
Dr. J. W. Torbett.
Dr. F. C. Walsh.
Dr. Joe Reuss.
Dr. Frank Paschal.

### The Social Malady.

BY DR. THEO. Y. HULL, SECRETARY STATE SOCIETY OF SOCIAL HYGIENE, SAN ANTONIO, TEXAS.

Many workers in discussing the subject of social hygiene speak of the "social emergency." I prefer to call it the social malady. A malady may be either a disease or a disordered condition. It may be acute or chronic in its nature. An emergency is a sudden condition calling for immediate action. The condition in which we find the social body is not of sudden origin. It is the most chronic of chronic conditions. It is as old as civilization. It has been regarded for centuries as a concomitant of civilization—a necessary evil. It is not a subject for sudden or precipitate action. It is a condition requiring calm, unprejudiced, thoughtful and persistent action. The danger that confronts the social hygiene movement today lies in the precipitate action of the enthusiast acting on impulse rather than reason. In every great movement for social betterment a period of what may be termed social hysteria precedes the more rational and substantial effort in which results are obtained. Roosevelt says that "every reform movement has its fringe of lunacy." Though the malady is serious and its continuance is fraught with grave consequences to the future, we can better afford to await an opportune time than by precipitate and ill-advised action defeat our purpose by arousing the antagonism of an uninformed public. We must bear in mind that our emancipation from the idea that man is something above and apart from the rest of creation is of recent occurrence. The idea that disease is a punishment inflicted upon us for violation of divine ordinances is not entirely eradicated today. We must, therefore, advance cautiously with due consideration for our environment. History is strewn with the wreckage of ideas prematurely advanced. Ancient Egypt and less ancient Greece furnish noted examples. Plato warned Greece of her danger, but the warning fell upon a public mind that did not comprehend it. Although we are fallen upon happier and more enlightened times, there still remains the preparation of the field if we may expect an abundant harvest. And this is our part in this movement, the preparation of the field, the sowing and the harvest will come later.

### UNDERLYING CAUSES.

Before attempting to plan and advise the remedy let us study the nature of the insidious malady that afflicts the body social. Is the disorder serious or not? What are the underlying causes?

On one occasion we asked an out-of-town patient the name of his home physician. He replied that when slightly ill he called Dr. A. When more seriously disordered, Dr. B, but when very sick he wanted "all the doctors in town." Now the question before us is not so much whether there exists a social disorder—that is usually conceded—but how serious the condition. Is a consultation necessary?

When we examine a patient in the consultation room or at the bedside we have three distinct objects in view—(1) to determine the nature of the disease, (2) to determine the extent of the pathological processes, and (3) to determine the kind of treatment indicated. We naturally infer that the patient desires relief. We now have the patient, the social body, one hundred million distinct living potential entities in the consultation room stripped of cosmetics, pretense and self-importance, and superfluous apparel—idleness, wealth, personal liberty. What are the symptoms?

### INDUSTRIAL AND LIVING CONDITIONS.

The first symptom of primary importance as indicating internal disorder relates to the industrial and living conditions of the people. This is manifest in an exaggerated idea of the importance of the commercial side of life, which contrasts strangely with the low valuation of human life shown in the National, State and municipal disregard of the health conditions of the masses. As a direct result of the long continued accentuation of commercial success, we have an increasing accumulation of wealth and power in the hands of the few, with a corresponding increase in poverty and dependence among the many.

If you care to investigate society you find that the very foundation on which the social superstructure rises is money. Your exclusive set is composed of the railroad magnate, the banker, the retired merchant, the cattleman and what not, provided he has wealth. It is a class distinction based on finances. When a family seeks to enter what is called society it is largely outside appearances that count. Physical and mental and moral worth is not so demanded as wealth. Society seemingly cares not a whit whether their ancestors were among the Jukes or the Edwards type.

### LEGISLATION ENACTED.

If you care to investigate the great mass of legislation enacted in our State Legislatures and even in our National Congress, you will find that the great bulk of it pertains to financial and commercial matters. Humanitarian legislation has little chance of enactment. We are quick to make property interest safe, but we are slow in making living conditions safe. Tuberculosis kills directly ten per cent of the people and indirectly ten to twenty per cent more. Since a large per cent of all individuals are infected and infection means lowered vitality if not actual disease, it very greatly decreases the efficiency of the people. Yet our State, like many others, will not take the first step for its eradication by enacting a compulsory notification law. This is not because the members of our legislative body do not believe in preventive medicine, but because their training has been such that they can only see its application in cases where there may be returns in dollars and cents. They are ready to appropriate the people's money when cattle and hogs or cotton and tobacco are concerned, because here they can see a money return on the investment. The A. and M. College is just now exploiting a hog cholera serum at State expense, but the legislative body will turn a deaf ear when money is asked for child welfare work. That is a work for charity. They also are willing to build insane asylums and kindred institutions, but they are absolutely averse to enacting laws that will in time diminish the supply of the feeble-minded, the insane and the criminal. They seemingly cannot comprehend legislation based on humanitarian ideas. Our National Congress is no better. Nearly all its time is consumed in considering matters that are intended to better the financial condition of the people, but when we ask Congress to establish a department of public health at Washington, it pleads economy. Notwithstanding it appropriated one and one-half millions for the work of the Bureau of Animal Industry and a larger sum for the operation of the Bureau of Plant Industry, it could not be induced to provide for the study of conditions which seriously affect the public health. Evidently our government does not consider man either plant or animal. This is only because we have taught our public men to think in terms of commerce, instead of in terms of human life and health. We plainly and inevitably put the dollar above the man. We can clearly understand the placard at the late Congress on Hygiene and Demography, hung where thousands could read, "Hogs Inspected," "Forest Protected," "Children Neglected." And this is true because we consider human life the cheapest commodity in America.

### DIRECTING EVOLUTION.

They tell us that eugenics is a dream, and that we cannot direct evolution. If any evidence were needed to show the possibility of producing the superman by directing the public mind in certain channels, the history of our industrial progress during the last half century will furnish an abundance. At the close of the Civil War the people were in desperate straits. Financial ruin was the rule. Poverty was on every hand and credit gone. The one great need of the country was money. The mind of the people became centered upon the one need. The brains of the country set about to secure relief. For the next thirty years the people thought largely in terms of commerce and finance, as the great mass of legislation during this period will testify. Tariffs and commerce, currency and banking, manufactures and transportation, agriculture and mining, invention and production of wealth occupied the center of the stage of human endeavor. As a result of this concentration of thought, we have vast accumulations of wealth and combinations of financial interests. We now have kings of finance, captains of industry and the like—men of brains whose ever tightening grip is felt and known in our legislative bodies and our National Congress, until today our great need is statesmen able to cope with the situation.

### EFFECTS OF POVERTY.

To every height there is a corresponding depth. In the depths, overshadowed by wealth and power, are the toiling thousands that constitute the most important element in the country, for from them come the bulk of the future citizenship. In one State alone there are over 300,000 unskilled laborers drawing a salary of less than $7.50 per week, and in another over 30,000 whose weekly earnings fall under $4.50. These toilers for the bare privilege of living form three-fifths of the wage earners of a great State. Yet these wage earners are supposed to have homes and families, but how and what? We cry out against child labor. It is a crime. We say it will dwarf them physically and mentally. It does. But they must live, and they cannot live unless they work, and they often die because they do work. We are horrified at the idea of prospective and expectant mothers drudging and toiling in factories and sweat shops. We say it is an injustice to the unborn. It is. But these toiling mothers must live and their offspring suffer because they toil to live. Yet these same offspring, starved before coming into existence and toiling to keep up existence, will be the citizens of the future. What can we expect? We say that the family is the social unit. What kind of a family? This intolerable wage is supposed to support a family of from three to eight. The making of good citizens out of these children can only begin when the parent has sufficient income to give them suitable environment.

This is a grave symptom of the social malady—one of which the Nation must some day take notice—a non-living wage—for out of such conditions come unrest, physical weakness, mental deficiency, moral perversion, disease and crime. It is a great canker-sore eating the vitality of the social body.

### UNDUE PREVALENCE OF DISEASE.

Another serious symptom of the social malady is the existence of an amount of disease entirely out of proportion to our supposed enlightenment in sanitary matters. This fact is brought home to us most forcibly by an absurdly and unnecessarily high infant mortality.

The modern study of disease shows that a very large per cent of illness is preventable. Pasteur held that it is within the power of man to rid himself of every parasitic disease. It is known that wherever hygiene is in use both the morbidity and the mortality of the people decrease. The period of life is lengthened directly in proportion to the sanitary precautions of the people. In Sweden and Denmark the average life is 50 years. In India it is 25. In some parts of Europe, 350 years ago, the average life was less than

20 years. Today it is 40 in the same regions. With increasing knowledge the truth is beginning to dawn upon our minds that the duration of life should be from 100 to 150 years, instead of the barely 45 of today. The fact that we do not live much longer is evidence of our failure to put in operation our knowledge of hygiene.

Viewed from a physical health standpoint the ideal life is one free from sickness and other disabilities, but the ratio of those who escape more or less lengthened periods of illness to those who do not is very small. If an individual were born of healthy parents, lived a normal life and observed the simple laws of hygiene, he should pass into old age imperceptibly and die a natural death, free from disease. As it is now natural death is almost unknown, and for each death from disease there is an average illness of two years. This means that the average individual suffers from some form of illness about two weeks each year.

In a city of 100,000 people, an average of about 1500 die each year. This varies in our American cities according to hygienic environment. The number actually varied in 1910 from 2260 in one city to 1100 in another. The actual causes of these 1500 deaths per 100,000 is worth our consideration. Typhoid fever was responsible for 23.5. In one city it rose to 86.7—poor sanitation, while in another it declined to 8.8—better hygiene. Yet we now know and have known for some time, that typhoid fever can almost invariably be prevented. In some European cities the rate is as low as 2. Measles takes 12.3, scarlet fever 11.6, and whooping cough, which parents too often think their children must have and health officers still neglect to quarantine, kills 11.6. Diphtheria still claims 21.4. Influenza is blamed, but is not guilty, for 14.4. Bright's disease, rapidly increasing because of faulty hygiene and abnormal living, 99. Diarrhea, due to improper food largely, 117.4. Heart disease due to a system overloaded with bacterial poisons, 141.5. Pneumonia, due to improper living and exposure, is charged with 147.7, though wrongly so. Tuberculosis, the arch fiend and foe of humanity, levies a tax of 160, and if those wrongly charged to influenza, heart disease and pneumonia, were charged to tuberculosis, it would exceed 200. Thus, in a city of 100,000 souls, eleven diseases, the active cause of most of which is well known, are responsible for 760 of the 1500 deaths. In this country 709,000 die from these eleven causes. It is well known that 70 per cent of these deaths are preventable. And why are they not prevented? It is simply because of the inertia of ages of indifference.

### INFANT MORTALITY.

Take the second factor, the high infant mortality, which exists in this country, largely unnecessary and partly criminal. Of all the 1,381,500 deaths in 1910, 19.2 per cent (261,250) were under one year (318,000 under two years). Of all deaths from diarrhea, 70 per cent were children under one year of age, and 29 per cent of all infant deaths during the same period were due to the same cause. Here improper food is the chief factor. Among the upper strata of human society the mother too often refuses to do the mother's part. In the lower strata the mother cannot do her part. She must live, and to live she must toil. Death is the penalty exacted of the offspring. Premature births and congenital debility wreck over 23 per cent more. And most of these are due to a crime which the law refuses to name and exacts no penalty. Yet these very factors which are active in destroying the young life are most active in the degeneracy of the race. To its greatest asset, the children, the State is indifferent, and indifference—lowered appreciation—is a striking symptom of degeneracy when apparent in a people, for the paternal instinct is the strongest of all instincts.

### FEEBLE-MINDEDNESS.

Another alarming symptom of our social malady—one that we ought not to pass as either harmless or hopeless and without remedy—is the existence of an unwonted and constantly increasing ratio of feeble-mindedness and insanity to the population. Insanity we usually class as a disease of the nervous system, but feeble-mindedness is not a disease. It is simply an early arrest of development in the nervous system, in which the gray matter of the brain is the chief sufferer. Normally the mental power of the individual should increase until added years too greatly enfeeble the whole organism. It should be an unfolding and growth of intellect, yet all down the line of years we find individuals dropping behind; some in early youth, some in their twenties, some a little later, but all have prematurely reached their limit and life thenceforth is a dull monotonous grind for existence. In degree it varies all the way for the Simple Simon of every town to the imbecile and idiot, from the child that could never keep up in school to the one that cannot be taught at all, from the one who can barely make a living when conditions are simple or primitive to the one that knows barely enough to eat.

Physical defects may often be removed, but mental defects are incurable and condemn the victim, for victim it is, to an intellectual sphere below that of the normal 13-year-old child. Feeble-mindedness in any degree means incompetency which makes it impossible to compete on equal terms with the normal in the struggle for life.

The number belonging to this class is much greater than we imagine. In England the known defective number half a million. In this country and every other country they constitute a serious burden on society, for out of these ranks come a great army of the unemployed, the petty thieves, the vagrants and their like, and those that largely fill our penal institutions, our almshouses and calmly eat the bread of charity.

Though feeble-mindedness is incurable in any degree, it is transmissible. Here is the great wrong that society permits to be inflicted upon itself. The social body seems never to realize the terrible significance of the fact. Few traits are transmitted with such certainty and regularity as mental defects. Two-thirds of all the inmates of institutions for the care of such have feeble-minded ancestors. It is said that 80 per cent of all cases are victims of a neuropathic inheritance. The other 20 per cent are victims of various diseases in the parents.

In this country and in every other country a large per cent of these are free to marry. It is an old saying that like begets like, or worse; and, what should concern the intelligent, the lower the social status, the higher the fertility. The defectives have much larger families than the normal.

INSANITY.

Turning to the second factor in this symptom of social distress, insanity, do we find it abating? It is estimated that there are 200,000 insane in this country today. In 1880, a third of a century ago, there was less than one-half this number, and what is more significant, the ratio between the number of the insane and the total population is increasing rapidly. In a little over two decades it rose from 183 per 100,000 population to 225. In England this ratio doubled in ten years. Like the feeble-minded, many insane marry and beget their kind in a greater proportion than the normal. From our youth orators have told us that "The hand that rocks the cradle rules the nation." Shall we be compelled to accept that other dictum by Rentoul "The hand that wrecks the cradle wrecks the nation?"

CRIME.

Closely related to the question of feeble-mindedness and insanity is that of crime, for it is more and more recognized that there exists a marked interrelation. Judging from the number of criminals who plead mental weakness in extenuation of their acts, a large per cent of all transgressions are of this type. It has long been a question whether criminals were not born more frequently than made. We now know that weakened mentality is inherited, and if this be linked with a criminal propensity, the latter will be transmitted as well. In the making of criminals, disease, by weakening self-control and perverting moral ideas, must be reckoned with. In fact, we are coming to speak of crime as a disease, and treat it accordingly. In the complexity of our present day life, and in the strife of competition, the weaker mentally and morally are forced to the wall and then begin with petty crimes, become vagrants or worse, and float around as human derelicts. With the increase of diseases of certain kinds, there goes an increase in crime. We may treat this question seriously or ignore it, but the fact remains that social disease and crime of the more serious kinds are rapidly increasing.

In 1850, when life was much less complex, there were 6737 prisoners in the United States. In 54 years the number was multiplied by 15. The ratio of prisoners to population in the former year was 29 to 100,000; in 1904 the ratio had risen to 125 per 100,000. From 1885 to 1889 there was an average of 38.5 murderers and homicides to the one million inhabitants, while from 1902 to 1906 the ratio had increased to 110. When we stop to consider that in this time the most serious of human crimes—murder—has increased threefold, the matter assumes a more serious aspect. The actual number of criminals in confinement at any one time compared to the total number of such offenders is small—probably not more than 10 per cent.

When we consider the mass of the feeble-minded, the criminal and the partially insane at large, their influence on social progress becomes evident. This influence is increased by the failure of our courts to protect the public against such, or to act as a deterrent force to the criminal. It is further increased by the system in our penal institutions which confirms the criminal instincts in the offender, instead of assisting in his reformation. The continuance of this symptom of the social malady means individual and racial decadence.

(To be continued.)

---

As a quick method of cauterizing the appendix stump, amputate the organ with knife or scissors dipped in pure phenol.—*American Journal of Surgery.*

